# Pesticides in Drinking Water—A Review

**DOI:** 10.3390/ijerph18020468

**Published:** 2021-01-08

**Authors:** Muhammad Syafrudin, Risky Ayu Kristanti, Adhi Yuniarto, Tony Hadibarata, Jongtae Rhee, Wedad A. Al-onazi, Tahani Saad Algarni, Abdulhadi H. Almarri, Amal M. Al-Mohaimeed

**Affiliations:** 1 Department of Industrial and Systems Engineering, Dongguk University, Seoul 04620, Korea; udin@dongguk.edu (M.S.); jtrhee@dgu.edu (J.R.); 2 Faculty of Military Engineering, Universitas Pertahanan, Bogor 16810, Indonesia; risky.kristanti@idu.ac.id; 3 Department of Environmental Engineering, Faculty of Civil, Planning and Geo-Engineering, Institut Teknologi Sepuluh Nopember, Surabaya 60111, Indonesia; adhy@its.ac.id; 4 Department of Environmental Engineering, Faculty of Engineering and Science, Curtin University Malaysia, CDT 250, Miri 98009, Malaysia; 5 Department of Chemistry, King Saud University, Riyadh 11451, Saudi Arabia; walonazi@ksu.edu.sa (W.A.A.-o.); tahanis@ksu.edu.sa (T.S.A.); muhemeed@ksu.edu.sa (A.M.A.-M.); 6 Department of Chemistry, College of Alwajh, Tabuk University, Tabuk 1144, Saudi Arabia; aalmarri@ut.edu.sa

**Keywords:** pesticides, water, fate, occurrence, advanced oxidation processes

## Abstract

The ubiquitous problem of pesticide in aquatic environment are receiving worldwide concern as pesticide tends to accumulate in the body of the aquatic organism and sediment soil, posing health risks to the human. Many pesticide formulations had introduced due to the rapid growth in the global pesticide market result from the wide use of pesticides in agricultural and non-agricultural sectors. The occurrence of pesticides in the water body is derived by the runoff from the agricultural field and industrial wastewater. Soluble pesticides were carried away by water molecules especially during the precipitation event by percolating downward into the soil layers and eventually reach surface waters and groundwater. Consequently, it degrades water quality and reduces the supply of clean water for potable water. Long-time exposure to the low concentration of pesticides had resulted in non-carcinogenic health risks. The conventional method of pesticide treatment processes encompasses coagulation-flocculation, adsorption, filtration and sedimentation, which rely on the phase transfer of pollutants. Those methods are often incurred with a relatively high operational cost and may cause secondary pollution such as sludge formation. Advanced oxidation processes (AOPs) are recognized as clean technologies for the treatment of water containing recalcitrant and bio-refractory pollutants such as pesticides. It has been adopted as recent water purification technology because of the thermodynamic viability and broad spectrum of applicability. This work provides a comprehensive review for occurrence of pesticide in the drinking water and its possible treatment.

## 1. Introduction

Pesticides are recognized as reagents for protecting crops against harmful pests and diseases in humans. The beneficial outcome of pesticides makes it become an important tool to maintain and improve the living standard of the global population. An average of 2 million tons of pesticides was used each year globally to confront weeds, insects and pests [1]. The conventional classification of pesticides based on the target species includes herbicides, insecticide, rodenticide, fungicide and so forth [2]. Herbicides and insecticides are the most common type of pesticide used, dominating 47.5% and the latter 29.5% of the total pesticide consumption [1]. The primary pesticide consuming countries including China, the USA, Argentina, India, Japan, Canada, Brazil, France, Italy and Thailand [2].

The pest control revolution has begun in the 1970s with the development of pesticides based on toxic heavy metals such as copper, lead, mercury and arsenic. This was followed by the discovery of dichlorodiphenyl trichloroethane (DDT) during World War II [1]. The use of DDT increased enormously due to its effectiveness against almost all pest species at low dosage. Because of the great use, adverse impact on the environment and mankind had become apparent as soon as DDT became popular. After DDT has been banned for agricultural and domestic use, a wide variety of synthetic pesticides has been produced, such as organophosphate and pyrethroid which are still toxic to the environment [3]. The continuous and excessive use of a wide range of pesticide eventually harm the non-target species and causes the pesticide residues to appear in many unexpected sites [4]. Under constant chemical pressure, pesticides had led to the development of resistant strains in which the pests and insects get immune to the pesticide [3].

The application of pesticides give rise to a range of benefits, including increased the quality and quantity of food and reduced insect-borne disease but raised the issues on the potential detrimental effects to the environment, including water resources. The associated environmental impacts are mainly due to the persistent and ubiquitous characteristics of various pesticides that posed havoc to the biodiversity [2]. The dissolution of pesticides depends on the nature of the compound, pesticide application techniques and climatic factors. The pesticides that are not readily degrading will either get accumulated in soils or mobilized from one site to another in the form of degraded products, with unknown toxicity to human health [2].

The occurrence of pesticides in the water body is derived by the runoff from the agricultural field and industrial wastewater. Despite the soil matrix that serves as a storage compartment of pesticide due to the high affinity of agrochemicals with soil, surface water resources like streams, estuaries and lakes, as well as the groundwater are susceptible to pesticide contamination because of the close interconnection of soil with water bodies. The low concentration of pesticides built up in water can get magnified through the food chain and enter aquatic organisms that are hazardous for human consumption [2]. Importantly, chronic exposure to pesticides through water ingestion can mimic the human body’s hormones that reduce body immunity, interrupt hormone balance, trigger reproductive-related issues, posing carcinogenic effects and reduce intelligence particularly towards the children under the body development stage [5].

This study reviewed the type of pesticide found in the water bodies, the sources of pesticide contamination, the fate and occurrence of pesticides in soil and water, the toxicity impacts on human health and the available treatment method of the pesticide-contaminated water. A recent study of pesticide-contaminated surface water that occurred in Tanjung Karang located at Kuala Selangor, Malaysia was also reviewed in this article.

## 2. Type of Pesticide Pollutants in Water

Pesticides are categorized into several distinct groups based upon their target species, such as insecticide, herbicides and fungicides being the most used in agricultural farmland and urban settings [1]. Table 1 reviewed the classes and chemical compounds of insecticide, herbicide and fungicide. Herbicides are weed-killing compounds and normally included in plant growth regulators. Insecticides are used in farmlands, food storage facilities or home garden to control insects. Fungicides prevent fungus infection in plants or seeds, which is usually applied before fungus present or after fungus infect the plant species [6]. Besides, the pesticide can be classified based on the mode of action on the pests such as destroying, mitigating and repelling reagent [3]. A more scientific way of pesticide classification is based upon their chemical composition. Table 2 outlined the main components of some of the common groups of pesticides.

The traditional pesticide before the 1940s was derived from the toxic heavy metal of arsenic, copper, lead and mercury. These chemicals are partially soluble in water; therefore, their residues present in foods are of far greater concern than in drinking water. Synthetic organic pesticides such as chlorinated hydrocarbons introduced during World War II have rarely contaminated the groundwater but tend to accumulate toxic concentrations in food chains. Some of the examples of chlorinated hydrocarbons are DDT, aldrin, endrin and chlordane, which are relatively insoluble in water but more likely to be chemically bound to the soil particles. Organophosphorus compounds such as diazinon and malathion are synthetic organic pesticides that are developed to replace the chlorinated hydrocarbon pesticides. Organophosphorus pesticides are still highly toxic to humans but their ability to decompose rapidly in the environment reduce their occurrence in groundwater. Carbamate pesticides are also being introduced to replace chlorinated hydrocarbons. The active ingredients of carbamate pesticides are not likely to be adsorbed to soil particles, therefore these compounds may have made their way into surface waters [11].

## 3. Sources and Fate of Pesticides in Water

The detectable concentration of pesticide in surface waters and groundwater found in agricultural and urban land use areas [12]. Pesticide moves into water bodies via point source and nonpoint source. Point source that originates from a fixed site including chemical runoff during improper storage, loading, disposal as well as the misapplication of pesticides to water bodies. A direct movement of pesticides into groundwater is a common type of point source pollution, in which the pesticides enter the water wells result from pesticide spills and improper disposal of pesticides. Urban use of insecticide is considered as a point source pesticide in surface waters. The non-point source is the movement of pesticides from large areas across the watersheds and eventually reached the water bodies over the time. Non-point sources of pesticide originate from the agricultural field initiate by the runoff and erosion events, leading to the gradual leaching of pesticides into the ground and surface water [13].

Contamination of pesticides in water is caused by the persistent chemicals of pesticides released from agricultural activities, urban use and pesticide production factories. Farmers is the key users of pesticide that apply an enormous amount of pesticide to protect and increase crop yields. Besides, the wood treatment industry uses an enormous amount of insecticide to treat the raw material. Depending on the pesticide’s characteristics, chemical compounds from the pesticide applied to the preserved material tend to be released into the environment, becoming one of the sources of pesticide contamination in surface waters. Despite the great use of pesticides in the agricultural sectors, the urban use of pesticides mainly in-house gardening for pest control is an important source of pesticide contamination in water. The insecticide is detected more profoundly in urban settings than other types of pesticides such as herbicide and fungicide [10]. Since the green revolution, increase consumption of pesticides led to the active production of pesticide formulations, which increase the pesticide manufacturer around the world [6]. Inevitably pesticide leaching processes throughout the pesticide manufacturing processes as well as in the dumping site and wastewater effluents contribute to point-source pesticide contamination. To summarize, pesticides in surface waters sourced by the run-off event, atmospheric deposition event, wastewater discharge and spills event, while pesticide in groundwater sourced by the pesticide-treated field, waste disposal site and pesticide manufacturing sites.

**Figure 1:**
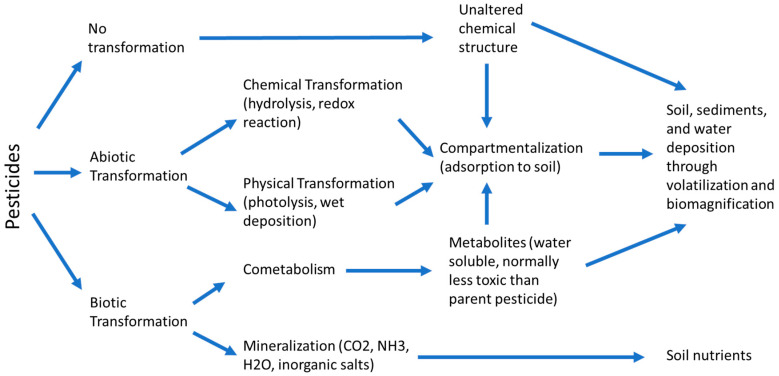
Fate of pesticide.

The study on the fate and transport of pesticides is important for knowing their circulation in the biosphere. Pesticides meet a variety of fates after being applied on Earth and Figure 1 showed the general picture of their fates in the environment. The pesticide that is not taken up by plants will either be retained in the soil or subjected to degradation into other chemical forms. Soluble pesticides will be carried away by water molecules especially during precipitation events by percolating downward into the soil layers and eventually reaching the groundwater. Otherwise, those insoluble chemicals tightly bound to soil particles accumulate in the topsoil layer, which has a high possibility subjected to runoff and erosion to surface waters, contaminating lakes, stream and river with pesticides. Pesticides are most susceptible to runoff immediately after the application on the soil surface between 0.25 to 0.85 cm from the soil surface [13]. Pesticide contamination in water also contributed by the volatilized pesticides in the atmosphere, in which they redeposited in the rain during the rainfall event and then enter the surface water bodies and soil. However, this pathway is relatively insignificant. In general, pesticides enter the hydrological system mainly via surface loss and leaching through soil layers, whereby the degree of pesticide contamination in water is affected by the properties of pesticide, characteristics of soil, site conditions, as well as the application and management practices of pesticide [14].

The potential for surface loss and leaching into groundwater is determined by the characteristics of pesticides such as the half-life, solubility and adsorption capacity of the pesticides. Since most pesticides are organic compounds, they typically undergo degradation through microbial, photochemical or chemical reactions. Microbial degradation including the mineralization process in which pesticide breaks down into carbon dioxide and co-metabolization where microbial reaction transforms pesticide into other chemical forms. Photochemical degradation of pesticides is called photolysis in which the pesticides decomposed in the presence of ultraviolet (UV) light. Chemical degradation of pesticide occurs via redox reaction and hydrolysis with air, water and other compounds exist in soil compartments. Pesticides with a low biodegradation rate have a long half-life and tend to persist in the environment that potentially contaminate the water sources. Besides, pesticide degradation processes produce metabolites, inorganic end-product and transformants which can have either lower or higher toxicity than the parent pesticide. Moreover, the mobility of the pesticide is governed by the adsorption capacity and solubility of the pesticide. Pesticides that are strongly adsorbed to soil are less likely to infiltrate downward the soil profile but can easily be carried by eroded soil particles via surface runoff and eventually reached surface water [14]. For pesticide having low degradation rate, weak adsorption capacity to soil particles and high solubility that is greater than 30 mg/L can potentially leach and dissolve in water. Among the pesticide used, atrazine which is normally used as an herbicide is recognized as a highly potential leach compound into the groundwater due to its high persistency. Cyanazine has a short half-life, therefore lower leaching potential. Methyl parathion is another low leaching potential pesticide because of its high adsorption capacity to soil particles and lower persistency. The 2,4-D is a water-soluble pesticide able to rapidly break down by biological action and therefore is less likely to accumulate in soil and has less persistency [15].

## 4. Occurrence of Pesticide and Health Effect

The occurrence of pesticides in specific environmental compartments, such as in soils and streambed sediment, groundwater and surface water is a widespread issue [16]. Distribution of a range of pesticides in streams and groundwater largely depends on the land-use settings and characteristics of the hydrologic system with consideration of the past and present use of pesticides. Pesticide detected most frequently in streams and groundwater were those in most use and with the compound characteristics of high mobility and persistence in the hydrologic system. 

Based on the National Water-Quality Assessment (NAWQA), pesticides are found more often in surface waters than groundwater, being 25 pesticides detected more than 10% of the time in surface waters and 2% of the time in groundwater of various land-use setting in agricultural, urban and mixed land use [12]. This proves that the occurrence of pesticides in surface waters is prevalent because of the direct and rapid overland mobilization of pesticides via surface runoff. Groundwater that is less vulnerable to pesticide contamination can be explained by the slow water infiltration rate through the soil into the aquifer. However, the extended travel time enables the pesticides to undergo transformation, dispersion and sorption that make contamination of groundwater more difficult to recover once it is contaminated. Of the 25 pesticides, 11 of them are herbicides that are widely applied in the agricultural field, 7 are herbicides used extensively in urban settings and 6 are insecticides applied in both agricultural and urban settings [12]. In undeveloped areas, detected pesticide in surface water and shallow groundwater is least often. In mixed land-use settings, the frequency of pesticide occurrence detected at stream draining watershed is similar to agricultural or urban settings because of the contribution of pesticides from multiple sources. Similarly, the detection frequency in shallow groundwater is prevalent over the major aquifers. According to the investigation, the pesticides that occurred most frequently in the streams and groundwater are the five agricultural herbicides—atrazine with its degradate, deethylatrazine, metolachlor, cyanazine, alachlor and acetochlor, the five non-agricultural herbicides—simazine, prometon, tebuthiuron, 2,4-D and diuron, as well as the 3 most extensive use insecticide—diazinon, chlorpyrifos and carbaryl [12]. For comparison, the insecticide was found more frequently in the urban stream than urban groundwater and also found in a higher concentration in comparison to agricultural settings.

Historical use of pesticides with their degradates and residues such as organochlorine is mostly found in soil, sediment and cell tissue of biota [12]. Review of a largely agricultural country of China, the history used of organochlorine in agricultural activities led to the different level of pesticides contamination in the groundwater, which is mainly driven by the extreme hydrogeological condition. The leaching of pesticides as well as their metabolites downward from soil surface had contaminated the shallow basins in China [17].

Despite the great importance of pesticides in maintaining good quality and protecting the crops or raw materials, they pose a high degree of concern in human health because of the tendency of pesticide to bioaccumulate in the human cell membrane which interrupts the body functioning system. Humans are exposed to pesticides in water mainly through dermal contact and ingestion [18,19]. Pesticide exposure has been proven to result in immunosuppression, hormone disruption, reduce intelligence, reproductive distortion and cancer. Impacts of pesticide exposure to humans can be categorized into acute health problems and chronic health problems. Chronic health problems encompass neurological effects such as onset Parkinson’s disease, reduce the attention span, memory disturbances, reproductive problems, disrupt infant development, birth defect and cancer. Acute health effects depend on the pesticide toxicity and the most common effects are reduced vision, headaches, salivation, diarrhea, nausea, vomiting, wheezing, coma and even death. Moderate pesticide poisoning leads to mimic intrinsic asthma, bronchitis and gastroenteritis [18].

In Malaysia, there is limited data that documented the effects of pesticides on human health. The study on utilizing biological markers to associate the effects of pesticide exposure to human health would be useful to evaluate the health risk [19]. A reviewed study by Samsuddin et al. documented that populations that are chronically exposed to low dose mix-pesticide are more likely to have cardiovascular diseases [20]. Another article revealed that the endosulfan led to the overexpression of stromelysins, a protein from the metalloproteinase family, which in turn degenerated the proteins involved in atherosclerosis progression [21]. Besides, few works have reviewed whether pesticide exposure could reduce semen quality, lower the sperm count and change the sperm’s morphology [21,22]. Literature also reported that farmers had a high chance of inducing prostate cancer and allergic or non-allergic asthma due to the frequent exposure to the chlorinated pesticide [23,24]. In animal studies, the genotoxicity effect of exposing organophosphate to orang-asli children was studied, which is observed through the changes in comet tail length [25,26]. Other animal study also found that rats exposed to organophosphate resulted in testosterone and hormone disruption in the testis [27].

As a measure to protect public health, guideline levels for pesticides in drinking water have been implemented by national governments. There are several guideline values, where few of them are issued by World Health Organization (WHO), the United States, Australia, the European Union and Japan. The guideline values may differ based on the socio-economical, dietary, geographical condition and industrial conditions [28]. Table 3 showed the guideline value for a certain number of pesticides in drinking water issued by WHO aimed for a water quality that is suitable for long-term consumption. These guideline values were made available for the use of regulatory authorities.

## 5. Pesticide-Contaminated Water Treatment Method—Advanced Oxidation Processes

The conventional method of pesticide treatment processes encompasses coagulation-flocculation, adsorption, filtration and sedimentation, which rely on the phase transfer of pollutants. Those methods are often incurred with a relatively high operational cost and may cause secondary pollution such as sludge formation [29]. Besides, indiscriminate use and the presence of a wide range of pesticide formulation available around the globe make the compound of pesticide in water harder to be removed. Therefore, alternative treatment processes are required to seek a long-term and feasible method to treat pesticide-contaminated water.

Advanced oxidation processes (AOPs) are recognized as clean technologies for the treatment of water containing recalcitrant and bio-refractory pollutants such as pesticides. It has been adopted as recent water purification technology because of the thermodynamic viability and broad spectrum of applicability [29,30]. The main concept of AOPs in the water treatment process is based on the in-situ generation of highly reactive hydroxyl radicals that indiscriminately oxidize a wide range of recalcitrant organic pollutant for complete mineralization of organic contaminants to carbon dioxide, water and mineral salts and capable of transforming the compound of pesticide into more biodegradable species [31,32]. Hydroxyl radicals can be produced from different pathways using a combination of oxidants, catalysts and ultraviolet irradiation and this makes the classification of AOPs based on the source of generation of hydroxyl (OH) radicals [33]. Table 4 showed some of the combinations of AOPs.

The integration of several AOPs into a sequence of complimentary water treatment processes is a common method to yields a more biodegradable effluent which can be further treated by the conventional biological process to reduce the reagent consumption and thus more economical in comparison to AOPs alone [32]. Based on previous studies, AOPs are recommended as pre-treatment steps to convert the pesticide to a more biodegradable intermediates, then followed by a biological treatment process to convert them into biomass, carbon dioxide, hydrochloric acid, biogas and water. This is mainly due to the inefficient removal of bulk chemical oxygen demand (COD) by AOPs to a level below the regulation standard for some recalcitrant pesticide compounds [29,34,35]. An article by Quiroz et al. also documented the extensive use of AOPs to treat pesticide-contaminated water [33].

In the field of AOPs, Fenton reactions have been widely studied for the remediation of pesticide-contaminated water because of the faster rate of pollutant removal and its ability to completely mineralized a wide variety of organic compounds [32,36]. In the Fenton process, Fenton’s reagent, which is prepared by adding iron salts as a catalyst in the hydrogen peroxide solution to generate strong hydroxyl radicals at an acidic condition. The general mechanisms of the Fenton process are shown in Table 5. It is worth noting that the depletion rate of Fe^2+^ is comparatively higher than the regeneration rate of Fe^2+^ from Fe^3+^ as illustrated in Table 5 Reaction 2. Because of this, the addition of Fe^2+^ or FeSO_4_ in the medium presence with hydrogen peroxide is necessary for continuous Fenton reaction to take place, which is not economical. Moreover, adding more Fe^2+^ result in more iron sludge produced that need to be handle in a proper way to avoid secondary pollution [37]. The drawbacks can be overcome by including the ultraviolet irradiation in the Fenton system, which is commonly known as the photo-Fenton process. The presence of UV visible irradiation with Fenton reagent enhance the process efficiency because of the capability to produce an additional source of hydroxyl radicals through photolysis of hydrogen peroxide and the photoreduction of Fe^3+^ to Fe^2+^ as illustrated in Table 5 Reaction 3, which subsequently increase the hydroxyl radicals yields and reduce the amount of Fe^2+^ required in Fenton reaction [38]. The optimum condition of the Fenton system has been studied which focusing on the pH, Fe^2+^ dose and the H_2_O_2_ dose [31]. From the study, the pH of the system needs to be kept at 3, because this is the optimum condition for the decomposition of hydrogen peroxide to produce hydrogen radicals and prevent the scavenging of hydroxyl radicals via dissociation and auto-decomposition of hydrogen peroxide as illustrated in Table 5 Reaction 5. Also, a lower pH value that is below or equal to 3 inhibits the occurrence of iron precipitation which enhances the UV radiation transmission into water. The hydrogen peroxide dosage depends on the COD of water, where the optimum COD: H_2_O_2_ ratio is 1:2.2 and 1:4.4 for photo-Fenton and Fenton treatment, respectively. For the Fe^2+^ dosage, the optimum H_2_O_2_:Fe^2+^ ratio is 50:1 and 100:1 for photo-Fenton and Fenton treatment, respectively [31]. As can be seen, photo-Fenton treatment reduced the consumption of H_2_O_2_ and Fe^2+^ to half in comparison to the Fenton process, which proved that the photo-Fenton process is more economical, more efficient in removing pollutants and produce less sludge. Another literature study by Oller et al. reported that the photo-Fenton reaction successfully eliminated six targeted water-soluble pesticides that are ineffectively removed by the TiO_2_ catalytic process [39]. However, the major drawbacks of the photo-Fenton process are the periodic addition of hydrogen peroxide that increase the operational cost and the use of UV visible light sources [38].

Moreover, heterogeneous photocatalysis is another appealing option to restore water contaminated with pesticide, that involves the use of solid photocatalyst to form a colloidal suspension under sunlight radiation to remove toxic substance in water [33]. The heterogeneous photocatalytic degradation has been proved to be able to remove a wide range of pesticides and the common pesticides being tested are triazine, thiocarbamide, phosphorated and chlorinated pesticide [33]. The solid catalyst act as an active site for adsorption of reactants and desorption of products. TiO_2_ is the most common semiconductor used as a photocatalyst because of the cost-effectiveness, high stability, non-toxicity and unique photocatalytic efficiency [40]. Table 6 summarized the reaction mechanism involves in the heterogeneous photocatalysis reaction. Because TiO_2_ has a bandgap energy of 3.2 V, the adsorption of UV light that is equal or greater than the bandgap energy of the semiconductor is required to activate or induce the charge separation. After excitation, the electron will move from valence band to conduction band, forming electron-hole pairs that act as electron donors or acceptors for the molecules that are in contact with the semiconductor [41]. On the surface of the semiconductor, the generated electron-hole pairs undergo redox reactions in the aqueous solution to produce hydroxyl radicals [40]. The reactive radicals will then attack the pollutants in the solution. This process gradually decomposes the contaminant, avoiding the residue and sludge production, thereby reducing the probability of secondary pollution. Besides, the catalyst remains unchanged during operation, therefore no consumable chemicals are required. Moreover, the contaminant is strongly adsorbed to the surface of the solid catalyst which make the photocatalytic process capable of removing contaminants effectively even at a very low concentration of contaminants in solution and this saves the water production cost [41]. However, this process relies on the UV absorption for activation and only 5% of solar irradiation falls within the UV range, resulting in lower production efficiency of reactive radicals. The study to improve the performance of TiO_2_ has been studied extensively, which is through the doping of TiO_2_ with foreign ions to narrow the TiO_2_ bandgap energy, thereby facilitates the generation of reactive radicals under visible light [38].

## 6. Case Study of Pesticide Contamination in Asian Countries

### 6.1. Malaysia

Tanjung Karang located in Kuala Selangor is a popular rice cultivation land in Malaysia. Pesticides are enormously applied in the rice cultivation land to increase crop yields and reduce crop damages. The consequence of the continuous and increasing use of pesticides has inevitably led to the contamination of the Tengi River, which is the source of the drinking water supply for a conventional drinking water treatment plant (Sungai Sireh Drinking Water Treatment Plant) and paddy irrigation. Based on the recent investigation by Elfikri et al. [42], eleven pesticides were detected in the river with concentrations ranging between 2.7 ng/L to 4493.1 ng/L and four pesticides detected in the finished water from the treatment plant with concentrations ranging between 5.2 ng/L–56.6 ng/L. The most frequently detected pesticides along the sampling site were imidacloprid with the highest detection level of 57.7 ng/L at downstream, which commonly used as insecticides and tebuconazole at a maximum detection level of 512.1 ng/L at the middle stream, which commonly used as fungicides in the rice cultivation land. This is attributed to the frequent application of the two pesticides, which are known to be applied every 15 days after the rice cultivation as protective measures by farmers [42]. The concentration of pesticides increases from the upper stream to the lower stream of the river. The Middle stream of the sampling point was detected with the highest concentration of propiconazole (4493.1 ng/L), followed by difenoconazole (1620.3 ng/L) and buprofezin (729.1 ng/L). Downstream of the Tengi River was reported to be the most polluted site with the greatest number of compounds pesticide being detected. Among the pesticide detected in the downstream river, propiconazole was among the pesticide compounds detected with the highest average concentration of 260.81 ng/L [42]. The higher detection level of pymetrozine might be due to its characteristics such as high-water solubility, slow degradation, non-volatile and exhibit a low adsorption capacity to soil particles. Besides, downstream rivers become the hotspot of a wide range of pesticides because of the release of irrigation water from paddy farms and oil palm plantations to the downstream after the sluice is opened.

The drinking water treatment plant that uptakes the water from the Tengi River in supplying potable water resources to the population in Tanjung Karang and Sekinchan. The conventional water treatment method, the filtration-coagulation-flocculation-sedimentation process, is adopted in the water treatment plant. As a safety consideration, it is necessary to investigate the pesticide contaminant removal efficiency of the water treatment process by the treatment plant to ensure safe drinking water. Before the water treatment process, nine pesticides with a maximum detection level of 392.8 ng/L were detected in water samples. After the combined water treatment process, all the targeted pesticide compounds were found to be less than 0.1 microgram/L which is below the standard level regulated by European Health-Based Chemical Standards [42]. The finished water test had found the four pesticides (pymetrozine, tebuconazole, propiconazole and buprofezin) were inadequately removed by the treatment process, with 23% of imidacloprid, 14% of buprofezin and propiconazole, as well as 12% of tebuconazole remain in the finished water. The five pesticides (pymetrozine, tricyclazole, chlorantraniliprole, azoxystrobin and trifloxystrobin) not detected in the finished water were completely removed by filtration, which is a process after the coagulation-flocculation processes [42].

The authors furthered their study to investigate the ingestion risk on the population of 510 in Tanjung Karang, who consumed the finished water supplied by the water treatment plant through the questionnaires survey [42]. It is generally known that chemicals with an octanol-water partition coefficient (log Kow) between 2–4 absorb well through the skin and log Kow exceeds 5–6 tend to bioconcentrate in the lipid membrane [43]. Therefore, the health impacts studied in this article focused on the consequences of chronic exposure to the four pesticides detected in the finished water, which is specifically on non-carcinogenic health risks. Hazard quotient (HQ) is a parameter to justify the level of non-carcinogenic risk by considering the daily exposure dose to the reference dose of target pesticide compound according to United States Environmental Protection Agency (USEPA). An HQ less than one specifies no significant risk, otherwise specifies significant non-carcinogenic health risks. The HQ results of the four target pesticides were less than 1, signifies daily ingestion of water supply from the treatment plant will have no significant chronic health risks. The hazard index (HI) is also determined for different age groups, classified into kindergarten, primary school, secondary school, adult and elder. The HI for all age groups is less than one, indicate no significant non-carcinogenic health risk upon the exposure to the combination of 4 target pesticides in the treated water. On the other hand, the young age groups were exposed to the highest level of targeted pesticide in comparison to the higher age groups because of the greater water consumption by the young age population [42]. Because the concentration of pesticides could vary according to many factors such as paddy farming season and the selection of pesticide by farmers, advanced water treatment processes that perform higher pesticides removal efficiency in water are recommended in water treatment facilities to guarantee the safest water supply to the population in Tanjung Karang and Sekichan [42].

Another study reported the concentration of selected organochlorine and organophosphate pesticides in the Selangor River in Malaysia. The organochlorine pesticides detected were lindane, heptachlor, endosulfan, dieldrin, endosulfan sulfate, DDT and DDE whereas for organophosphate pesticides, they were chlorpyrifos and diazinon. The concentration range of detected residual pesticides in the Selangor River, Malaysia during 2002–2003 were 10.1 ng/L for lindane as the lowest pesticide concentration and 1848.7 ng/L for endosulfan as the highest pesticide concentration. The study has revealed that agriculture, urban and industrial activities in the state of Selangor coupled with high population growth have caused deterioration in its river water quality. It was found that pesticides detected in raw river water intake were not removed by conventional water treatment process [44].

### 6.2. Japan

In Japan, paddy fields contribute significantly to the pesticide contamination of Japanese rivers because they account for about 50% of Japan’s agricultural lands of 4.8 million ha. This non-point source pollution of rice pesticides is of great concern because river water accounts for about 70% of drinking water sources in Japan. The Shinano River, known as the Chikuma River in its upper reaches, is the longest and widest river in Japan and the third largest by basin area. It is located in northeastern Honshu. Among the total of 53 chemicals found, 22 were herbicides, 15 were insecticides, 11 were fungicides and 5 were metabolites. The concentrations of chemicals found ranged from 3 ng/L (bromobutide) to 8200 ng/L (isoprothiolane). The transfer of pesticide in Shinano River water to the sea means pesticide entering rivers will also affect marine organisms, especially fish and may subsequently affect humans consuming marine organisms [45]. The Kurose River, a river in Hiroshima Prefecture, is approximately 43 km long. The Kurose River flows through urban and agricultural areas on the Kamo Plateau, including Higashi-Hiroshima city, before entering the Seto Inland Sea. The Kurose River has a surface area of approximately 250 km^2^. Agricultural runoff and wastewater containing industrial and household pollutants enter the Kurose River. The water flowing from the Kurose River into the Seto Inland Sea will, therefore, transfer OPs that may affect aquatic organisms, especially fish, in the Seto Inland Sea and these OPs may subsequently affect humans. The concentrations of pesticide found ranged from 2.8 ng/L (fenarimol) to 1194 ng/L (diazinon). Cyanazine was the most frequently detected pesticide, followed by simetryn and then diazinon. The presence of simetryn and isoprothiolane was largely attributed to rice paddy farms, whereas diazinon was associated mostly with vegetable farms and orchards. The diazinon and isoprothiolane patterns were consistent with their use of controlling insects and fungi in the prefecture [46]. The high contamination of Kurose River water with pesticides compared to other rivers may be due the runoff of pesticide residues from a big agricultural area in Hiroshima prefecture, including Higashi-Hiroshima City, which is cultivated with rice and vegetables crops in which high amounts of pesticides were used for controlling pests [47].

### 6.3. China

The huge utilization of pesticides has reduced the existence of pests and has increased crop production in China. However, the application of pesticides at such high concentrations has induced residual pesticides in soil. Huangpu River basin has been an agriculturally developed region since ancient times due to abundant radiation and heat, heavy rainfall, numerous rivers and lakes and fertile soil. The water quality of the Huangpu River was severely affected by agricultural non-point source pollution as well as industrial and urban sewage, which resulted in the water quality falling to Class III–IV, impacting the basic ecological functions and service of the river. Among the total of 29 pesticides analyzed, 18 were present in every sample taken from the Huangpu River. The concentration of target pesticides in water samples ranged from <LOQ (buprofezin) to 607.30 ng/L (carbendazim). The concentration of carbendazim is high because it widely used as a broad-spectrum fungicide in the cultivation of crops, such as rice, wheat and cotton [48]. The Dongjiang River basin is located in southern China with a total area of 35,636 km^2^. It flows through one of the China’s most developed provinces, Guangdong. The main land use types in Dongjiang River basin are mixed forest, agriculture land and orchard, composing approximately 66%, 17% and 5% of the total area. Among the total of 3 pesticides (Chlorpyrifos, triazophos and isoprothiolane) tested, the highest concentration of chemicals found was 279.51 ng/L (isoprothiolane). Isoprothiolane is an organosulfureous fungicide used to prevent diseases of paddy rice, with a usage of 35,320 kg in the study area [49].

### 6.4. India

River Yamuna, one of the major rivers of India with a total stretch of 345,843 km^2^, passes through Haryana state along its eastern border. However, due to high-density population growth and fast industrialization, Yamuna has become one of the most polluted rivers in the world. The concentration of Hexachlorocyclohexane and DDT at different sites of the river ranged between 12.76–593.49 ng/L and 66.17–722.94 ng/L, respectively. In canals the values were found between 12.38–571.98 ng/L and 109.12–1572.22 ng/L for Hexachlorocyclohexane and DDT [50]. The Gomti River, one of the major tributaries of the River Ganga originates from a natural reservoir in the swampy and densely forested area. The river serves as one of the major source of drinking water for the Lucknow City, the State capital of Uttar Pradesh with a population of about 3.5 million. Among the sample analyzed, 21 pesticide were present in river water and bed sediment taken from the Huangpu River. In the water of Gomti River, pesticide residues ranged between 2.16 to 567.49 ng/L and in the bed sediments it ranged from 0.92 to 813.59 ng/L. It was suggested that source of DDT contamination is from the aged and weathered agricultural soils with signature of recently used DDT in the river catchments. The results revealed that bed-sediments of the Gomti River are contaminated with lindane, endrin, heptachlor epoxides and DDT and may contribute to sediment toxicity in the freshwater ecosystem of the river [51]. The concentrations of pesticide detected in some rivers in Asia is summarized in Table 7.

## 7. Conclusions

The occurrence of pesticides in the water poses a deleterious effect on human health, where the effect magnitude depends on the solubility, half-life, adsorption capacity, biodegradability of the pesticide compounds. In the future, chemical pesticides will continue to perform a vital role in pest management. Despite evaluations of the efficacy, ease of use and cost of pesticides, the potential adverse effects of pesticides should be taken into consideration to achieve long-term sustainability pest management. Research in the field of pesticide development and technologies should be enhanced for compatible ecological based pest management. Assessment of pesticide residue management, the fate of pesticides and application technology would be useful for reducing the adverse health impacts from pesticides and its alternatives. With no justification for completely phasing out the chemical pesticide, pesticide users are recommended to replace the use of synthetic pesticides with bio-pesticide that exert a lesser environmental impact and also to ensure the correct application of pesticides in the agricultural system. Besides, Integrated Pest Management (IPM) is an ideal strategy for managing pests and insects in urban and agricultural settings that offer long-term prevention of pests by natural means. With the selective pesticide for backup in IPM, the usage of pesticides could be reduced to a larger extent, reducing the occurrence of pesticide compounds in water. As for safety measures, the water bodies in which pesticide compounds have been detected should undergo constant monitoring and potable water should undergo advanced water treatment processes if required.

## Figures and Tables

**Table 1:** Classification of pesticide based on target species.

Pesticide	Class: Substance
Insecticide [7]	Organochlorine: Endosulfan
	Organophosphate: Diazinon, Malathion, parathion, chlorpyrifos
	Carbamate: Aldicarb, carbofuran, carbaryl
	Pyrethroid: Deltamethrin, Fenpropathrin
	Neonicotinoid: Acetamiprid, thiamethoxam
	Phenylpyrazole degradate: Aldicarb sulfoxide, Endosulfan sulfate
Herbicide [8,9]	Triazine: Atrazine, cyanazine
	Chloroacetamide: alachlor, butachlor, dimethenamid, metolachlor
Fungicide [10]	Benzamide: Fluopicolide, zoxamide
	Carboxamide: Boscalid captofol
	Chlorinated hydrocarbon: Hexachlorbenzene
	Organophosphate: Edifenphos, iprobenfos
	Chlorophenyl: Dichloran, quintozene

**Table 2:** Pesticide classification according to chemical composition and provided with some of their general characteristics [4].

Group	Chemical Composition	Characteristics	Effects
Organochlorine (DDT, aldrin, lindane, chlordane)	Non-polar and lipophilic atoms including carbon, chlorine, hydrogen atoms.	Lipid soluble, toxic to variety of animals and long-term persistence.	Tend to accumulate in fatty tissue of animal, biomagnification effect via food chain.
Organophosphate (Malathion, diazinon, parathion)	Aliphatic, cyclic and heterocyclic possess central phosphorus atom in molecule.	Soluble in organic solvent as well as water. Less persistence than chlorinated hydrocarbons.	Tend to infiltrate into aquifer and reach groundwater. Affect central nervous system.
Pyrethroids (pyrethrins)	Alkaloid obtained from petals of plant species, namely, Chysanthemun cinerariefolium.	Less persistent than other pesticides, therefore safest to be used as household insecticides.	Affect nervous system.
Carbamates (Carbaryl)	Chemical structure based on alkaloid of a plant species, namely Physostigma venenosum.	Relatively low persistence.	Only killed limited spectrum insects but highly toxic to vertebrate species.
Biological (Becillus thuringiensis, Bt and its subspecies)	Microorganism, viruses and their metabolic products.	Applied against forest pests (butterflies) and crops.	Affect other caterpillars.

**Table 3:** Guideline value of pesticide level in drinking water [28].

Pesticide	Guideline Value (Microgram/L)	Pesticide	Guideline Value (Microgram/L)
Alachlor	20	Metolachlor	10
Aldicarb	10	Molinate	6
Atrazine	2	Pentachlorophenol	9
Bentazone	300	Permethrin	20
Carbofuran	7	Propanil	20
Chlordane	0.2	Pyridate	100
DDT	2	Simazine	2
Hexachlorobenzene	1	2,4,5-T	9
Isoproturon	9	Terbuthylazine	7
Lindane	2	Trifluralin	20

**Table 4:** Combination of advanced oxidation processes (AOPs) [33].

AOPs	Combination
Photocatalysis	UV/TiO_2_; UV/TiO_2_/H_2_O_2_
Fenton based	Fenton: Fe^2+^/H_2_O_2_
	Photo-Fenton: Fe^3+^/H_2_O_2_/UV
Ozone based	O_3_/H_2_O_2_; O_3_/UV; O_3_/UV/H_2_O_2_
Sonolysis	Ultrasound (US)/O_3_; US/H_2_O_2_; US/UV/TiO_2_
Electrochemical oxidation	ElectroFenton: Fe^3+^/H_2_O_2_(e^−^)
	SonoElectroFenton: US/Fe^3+^/H_2_O_2_(e^−^)

**Table 5:** Chemical reactions of Fenton/photo-Fenton process [37,38].

Reaction No.	Fenton/Photo-Fenton Reaction	Explanation
1	Fe^2+^ + H_2_O_2_ → Fe^3+^ + •OH + OH^−^	Chain initiation. Ferrous ions (Fe^2+^) catalysed the oxidation of hydrogen peroxide, generating hydroxyl radical.
2	Fe^3+^ + H_2_O_2_ → Fe^2+^ + H^+^ + HO_2_•	Mechanisms involve in the regeneration of Fe^2+^.
	Fe^3+^ + HO_2_• → Fe^2+^ + H^+^ + O_2_	
	Fe^3+^ + O_2_•^−^ → Fe^2+^ + O_2_	
3	Hν H_2_O_2_ → 2OH	Presence of UV visible irradiation in Fenton reaction increase the hydroxyl radical production and regenerate Fe^2+^.
	Fe(OH)^2+^ + hν → Fe^2+^ + OH	
4	R + H_2_O_2_ → P1	Reactive radical attaches the pollutants.
	R + •OH → P2	
5	Fe^2+^ + •OH → Fe^3+^ + OH^−^	Reactions that scavenge the hydroxyl radical.
	H_2_O_2_ + •OH → HO_2_• + H_2_O	

• Free radical.

**Table 6:** Chemical reaction of heterogeneous photocatalysis reaction [40,41].

Heterogeneous Photocatalysis Mechanisms	Explanation
Photocatalyst + *hν* → h^+^ + e^−^	Semiconductor absorb UV light producing electron-hole pairs.
h^+^ + H_2_O → ˙OH + H^+^ [Oxidation]	Reaction of holes in valence band with water molecules on the surface of catalyst produce hydroxyl radical. Reaction of electron in conduction band with oxygen generate superoxide radical, which then further reaction to produce hydroxyl radical.
e^−^ + O_2_ → ˙O_2_^−^ [Reduction]	
˙O_2_^−^ + H^+^ → ˙OOH	
2˙OOH → O_2_ + H_2_O_2_	
H_2_O_2_ + ˙O_2_^−^ → ˙OH + OH^−^ + O_2_	
h^+^ + OH^−^ → ˙OH	Other mechanisms involve in the production of hydroxyl radical.
H_2_O_2_ + *hν* → 2OH	
h^+^ + pollutant → (pollutant)^+^	Reactive radical attack or degrade the pollutant in solution.
Pollutant + (˙OH, h^+^, ˙OOH or O_2_^−^) → degradation product	

**Table 7:** Concentrations of pesticide in some Asian rivers reported in literature.

Country	Location	Concentration	Reference
Malaysia	Tengi River	2.7 ng/L–4493.1 ng/L	[42]
	Selangor River	10.1 ng/L–1848.7 ng/L	[44]
Japan	Shinano River	3 ng/L–8200 ng/L	[45]
	Kurose River	2.8 ng/L–1194 ng/L	[47]
China	Huang Pu River	ND–10,370 ng/L	[48]
	Dongjiang River	ND–279.51 ng/L	[49]
India	Yamuna River	12.38 ng/L–1572.22 ng/L	[50]
	Gomti River	0.92 ng/L–813.59 ng/L	[51]
